# Improving the Efficacy of Antimicrobials against Biofilm-Embedded Bacteria Using Bovine Hyaluronidase Azoximer (Longidaza^®^)

**DOI:** 10.3390/pharmaceutics13111740

**Published:** 2021-10-20

**Authors:** Elena Trizna, Diana Baidamshina, Anna Gorshkova, Valentin Drucker, Mikhail Bogachev, Anton Tikhonov, Airat Kayumov

**Affiliations:** 1 Institute of Fundamental Medicine and Biology, Kazan Federal University, 420008 Kazan, Russia; trizna91@mail.ru (E.T.); prosto-di@mail.ru (D.B.); 2 Limnological Institute of the Siberian Branch of the Russian Academy of Sciences, 664000 Irkutsk, Russia; kovadlo@lin.irk.ru (A.G.); drucker@lin.irk.ru (V.D.); 3 Biomedical Engineering Research Centre, St. Petersburg Electrotechnical University, 197022 St. Petersburg, Russia; rogex@yandex.com; 4 NPO Petrovax Pharma LLC, 123112 Moscow, Russia; info@petrovax.ru

**Keywords:** bacterial biofilms, enzymatic destruction of the biofilm, bovine hyaluronidase azoximer (Longidaza)

## Abstract

While in a biofilm, bacteria are extremely resistant to both antimicrobials and the immune system, leading to the development of chronic infection. Here, we show that bovine hyaluronidase fused with a copolymer of 1,4-ethylenepiperazine N-oxide and (N-carboxymethyl) -1,4-ethylenepiperazinium bromide (Longidaza^®^) destroys both mono- and dual-species biofilms formed by various bacteria. After 4 h of treatment with 750 units of the enzyme, the residual biofilms of *Staphylococcus aureus*, *Enterococcus faecalis*, *Escherichia coli*, *Pseudomonas aeruginosa* and *Klebsiella pneumoniae* preserved about 50–70% of their initial mass. Biomasses of dual-species biofilms formed by *S. aureus* and the four latter species were reduced 1.5-fold after 24 h treatment, while the significant destruction of *S. aureus–P. aeruginosa* and *S. aureus–K. pneumoniae* was also observed after 4 h of treatment with Longidaza^®^. Furthermore, when applied in combination, Longidaza^®^ increased the efficacy of various antimicrobials against biofilm-embedded bacteria, although with various increase-factor values depending on both the bacterial species and antimicrobials chosen. Taken together, our data indicate that Longidaza^®^ destroys the biofilm structure, facilitating the penetration of antimicrobials through the biofilm, and in this way improving their efficacy, lowering the required dose and thus also potentially reducing the associated side effects.

## 1. Introduction

Although rapidly expanding the use of implants, implanted artificial systems and invasive devices such as vascular or urinary catheters, ventilators, and heart valves helps to save the lives of millions of patients around the world, biofilm formation on their surfaces remains a common cause of in-treatment and post-surgery complications, inflammations and implant rejections [1,2]. Intravascular catheters and urinary catheters, being the two most commonly applied invasive medical devices, quite unsurprisingly also appear among the most common causes of nosocomially acquired infections [3,4,5,6,7,8]. In immunocompromised patients, even residential microflora being coupled with various nosocomial pathogenic bacteria may eventually cause catheter-associated infections, with the subsequent development of various diseases of the urinary tract, such as cystitis, pyelonephritis, bacteremia, urosepsis, prostatitis, epididymitis, and septic arthritis etc. [1].

*Escherichia coli*, *Pseudomonas aeruginosa*, *Klebsiella pneumoniae*, *Proteus mirabilis*, *Proteus vulgaris*, *Citrobacter* sp., *Staphylococcus aureus*, *Staphylococcus epidermidis*, *Enterococcus faecalis*, *Providentia rettgeri* and *Candida albicans* [9,10,11,12] are common urinary tract pathogens. Among them, *E. coli* is the most frequent agent causing about 80% of urinary tract infections in humans, as well as bacteremia associated with Gram-negative bacteria in hospitalized patients [9,13,14,15,16]. The above bacteria generally form rigid biofilms on both the inner and outer surfaces of implanted catheters, as well as on the epithelium of the urinary tract; in this way, they represent a common cause of chronic infectious diseases [17,18,19]. Depending on how long the catheter remains within the body, either monomicrobial (rather common for short-term catheters) or polymicrobial (rather common for long-term catheters) biofilms are often developed on their surfaces [6]. Biofilm formation on the urinary tract epithelium facilitates the further penetration of pathogenic bacteria into the renal tissue, which in turn often leads to chronic bacterial prostatitis and pyelonephritis [20,21]. Another negative outcome of the biofilm formation in the urinary tract is the formation of kidney stones as a result of the interaction between uropathogenic bacteria and the minerals of the urine [22]. Urease producers such as *Proteus* sp., *P. rettgeri*, *K. pneumoniae*, *S. aureus* and *P. aeruginosa* appear to be the most common microorganisms associated with the formation of kidney stones [16,20,23]. Urethral catheter surfaces are the common sites of bacterial biofilm formation, with explicit indications of *E. coli* biofilm fouling being observed already after 4–12 h of incubation. Continuous ambulatory peritoneal dialysis catheters are commonly infected with such bacteria as *S. epidermidis* and *P. aeruginosa*, which often cause complications of nephrological diseases as well [24,25,26,27].

Biofilm-associated infections are extremely difficult to treat due to the diffusional barrier formed by the biofilm matrix, which prevents the penetration of antimicrobials into the biofilm [11,28], resulting in up to 1000-times higher tolerance to antibiotics [20]. Moreover, while using combinations of antibiotics could lead to positive results when treating biofilm-associated infections, the intensive development of bacterial antibiotic resistance largely diminishes the efficacy of the available options [29,30].

To date, various strategies have been offered for targeting topical biofilms, focusing either on their destruction or on the prevention of their formation, or both [2,31,32,33,34,35,36,37,38,39]. Nevertheless, very few options are available for the treatment of urinary biofilm-associated infections. For example, modified catheters and implants based on hydrogel, Poly (Tetralfouroethalene) (PTFE) coatings, Polyzwitterions coatings, and Poly (Ethylene Glycol) (PEG) coatings could be useful for the prevention of fouling [2,40], while a number of electrophysical and electrochemical approaches targeting already-formed biofilms have recently been proposed [41].

One of the promising strategies for the destruction of already-formed biofilms is their enzymatic treatment. In numerous in vivo and in vitro studies, an efficient disruption of mature biofilms by various enzymes like DNase [42,43] and proteases (Ficin [35], Proteinase K [44] and aureolysin [45,46]), glycoside hydrolases (Pel, Psl [47], dispersin B [48], alginate lyase [49], cellulase [50] and extracellular levanase from *Bacillus subtilis* [51]) has been reported. Biofilm matrix lysis leads to increased bacterial susceptibility to antimicrobials, and thus also a considerable improvement of the latter [33,35,51,52]. Unfortunately, there is no universal enzyme efficiently targeting arbitrary biofilms due to the considerable differences in the composition of the proteins, polysaccharides, and extracellular DNA in the biofilm matrix depending on the bacterial species and their growth conditions [53]. Another drawback of this approach is that many enzymes are either not approved for clinical applications or only topical use is possible [52].

Several studies reported the contribution of bacterial hyaluronidases to the destruction of the biofilm matrix components, leading to subsequent cell dispersion [54,55]. Bovine hyaluronidase fused with a copolymer of 1,4-ethylenepiperazine N-oxide and (N-carboxymethyl)-1,4-ethylenepiperazinium bromide (Longidaza^®^) [56] is approved for application as a suppository, and has been successfully used in clinical practice since 2007 as a part of complex therapy for diseases accompanied by connective tissue hyperplasia associated with adhesive, scar, and fibrotic processes. Besides its positive effect on tissue recovery, several studies [57,58,59,60] have shown changes in the bacteriological profiles of the cervical canal, urinary tract and semen, suggesting the apparent effect of Longidaza^®^ on bacterial adhesion and biofilm dispersion.

Here, we show that Longidaza^®^ is capable of destroying biofilms formed by *S. aureus, E. faecalis, E. coli, P. aeruginosa, K. pneumoniae, S. marcescens* monocultures, as well as mixed biofilms formed by *S. aureus* and other bacteria. We show explicitly that the enzyme reduces the respective biofilm biomasses 1.5–2-fold and, being combined with antimicrobials, increases the efficiency of the latter against biofilm- and cell-clump-embedded bacteria.

## 2. Materials and Methods

### 2.1. Reagents and Enzymes

A commercially available Longidaza^®^ powder (NPO Petrovax Pharma LLC, Moscow, Russia), at 3000 international units per vial, was solubilized in nutritional broth and added to final concentrations of 85–750 IU/mL. The other chemicals were reagent grade, and were purchased from Sigma, St. Louis, MO, USA.

### 2.2. Bacterial Strains and Growth Conditions

A number of Gram-positive (*Staphylococcus aureus* ATCC^®^ 29213™ and *Enterococcus faecalis* clinical isolate) and Gram-negative (*Escherichia coli* MG1655, *Pseudomonas aeruginosa* ATCC^®^ 27853™, *Klebsiella pneumoniae* clinical isolate, *Serratia marcescens* clinical isolate) bacteria were used as the test organisms. A clinical isolate of *Enterococcus faecalis* was obtained from the Kazan Institute of Epidemiology and Microbiology (Kazan, Russia). *Klebsiella pneumoniae* and *Serratia marcescens* were obtained from the Institute of Medical Microbiology, Giessen, Germany. The bacterial strains were stored as a 50% glycerol stock at −80 °C, while they were maintained and grown on the Luria-Bertani medium (LB) during experiments. The modified Basal medium (BM) (glucose 5 g, peptone 7 g, MgSO_4_ × 7H_2_O 2.0 g and CaCl_2_ × 2H_2_O 0.05 g in 1.0 L tap water) was chosen for the biofilm assays [35,36,59]. The bacteria were grown under static conditions for 48 h at 37 °C to obtain rigid biofilms [36,61].

### 2.3. Antibacterial Activity

The MIC of the antimicrobials was determined by the broth microdilution assay in 96-well plates (Eppendorf, Hamburg, Germany) according to the EUCAST rules for antimicrobial susceptibility testing [62] in BM broth. The concentrations of the antimicrobials ranged from 0.25 to 512 µg/mL. The MIC was determined as the lowest concentration of an antibiotic for which no visible bacterial growth could be observed after 24 h of incubation. Furthermore, 1000-fold dilutions of the culture liquid from the wells without visible growth were prepared in BM broth to determine the MBC. The antibiotic’s concentration with no bacterial growth was considered as the MBC.

### 2.4. Biofilm Assays

The bacteria (2–9 × 10^6^ CFU/mL) were seeded in BM broth and grown under static conditions in 24-well TC-treated polystyrol plates (1 mL per well). After 48 h of growth, the old broth was exchanged with the new one, compounds of interest were added up to the final concentrations as indicated in the figures (see the X-axis labels for concentration values), and the incubation was continued for the next 4 or 24 h. Then, the supernatant was saved for further analysis, and the wells were washed several times with sterile phosphate-buffered saline (PBS) to remove the nonadherent cells. The obtained samples were subjected to either crystal violet staining [63] with modification [35], or a Congo Red depletion assay [64].

For the crystal violet staining, the liquid culture was removed after 4 or 24 h of incubation, and the plates were washed twice with PBS (pH 7.4) and dried overnight. Then, 1 mL 1% crystal violet solution (Sigma) in 96% ethanol was added per well, followed by 20 min incubation. Next, the crystal violet solution was removed and the plate was washed 3 times with PBS. After 30 min air drying, 1 mL 96% ethanol was added to re-solubilize the bound crystal violet, and the absorbance was measured at 570 nm with the microplate reader Infinite 200 Pro (Tecan, Männedorf, Switzerland). The biofilm biomass was expressed as a percentage of the residual biofilm, considering the optical density in non-treated wells as 100%.

For the Congo Red assay, the culture fluid was removed from the test wells and 500 μL Congo Red solution (80 μg/mL in LB) was added to the wells, after the biofilm was mechanically scratched from the surface, followed by 90 min incubation at 37 °C. Then, the plates were centrifuged for 5 min at 4400 rpm, and the supernatant was transferred to 96-well plates and measured on a Tecan infinite 200 Pro microplate reader (Tecan, Männedorf, Switzerland) at 490 nm. The results were expressed as the Congo Red (in optical units) uptaken from the solution.

In order to assess the effect of the antimicrobials on the viability of the biofilm-embedded cells, bacterial biofilms were grown under static conditions in the BM broth for 48 h at 37 °C, washed, and exposed to 1, 4, 16× MBCs antimicrobials (see MBC values in Table S1) for 24 h, either in the presence or in the absence of Longidaza^®^ (750 IU/mL) in fresh BM. The viability of the cells was assessed by MTT assay [65]. Briefly, the wells were washed twice with 0.9% NaCl to remove non-adherent cells. The MTT solution (1 mg/mL in PBS) was added into the wells with the biofilm, followed by the mechanical removal of the biofilm from the surface and incubation at 33 °C until formazan crystals could be observed in the control (non-treated) wells. Next, the samples were centrifuged for 5 min at 4400 rpm, and the liquid was replaced with dimethyl sulfoxide (Sigma-Aldrich, St. Louis, MO, USA) and incubated for 15 min at 33 °C to dissolve the formazan crystals. The absorption was measured on a Tecan Infinite 200 Pro at 570 nm.

In order to assess qualitatively the viability of the bacteria in the biofilm and cell-clumps after their exposure to the antimicrobials, the resazurin assay was performed. Briefly, mature biofilms were treated with antibiotics and Longidaza^®^ as described earlier. After 24 h of incubations, the detached cells in the culture fluid were transferred to new plates, harvested by centrifugation for 5 min at 4400 rpm, and resuspended in 160 µL 0.9% NaCl. The biofilms were washed once with 0.9% saline and destroyed mechanically in 160 µL 0.9% NaCl. After that, 40 mL resazurin solution (0.1 mg/mL) was added to the samples and incubated for 15 min at 30 °C until the pink color could be observed in the non-treated samples. The blue color indicated the death of the bacterial cells.

### 2.5. The Quantification of the Matrix Composition

The content of proteins and polysaccharides in the biofilm matrix was assessed by biofilm staining with the fluorescent dyes Sypro Orange (ready to use ×1000 solution), ConA-TMR (500 µg/mL), and Calcofluor White M2R (CFW, 1 mg/mL). All of the dyes were purchased from Sigma. The 48 h-old biofilms were treated with Longidaza^®^ for 24 h. Next, the culture liquid was removed from the wells, washed once with 1× PBS solution, and the dyes were added to the biofilms (100 µL per well) followed by 15 min incubation at 37 °C. Then, the wells were washed with 1× PBS, filled with 100 µL PBS, and the fluorescence was measured on a microplate reader Tecaninfinite 200 Pro (Männedorf, Switzerland) at the desired wavelengths (see Table 1).

### 2.6. Scanning Electron Microscopy (SEM)

The structure of the biofilms after treatment with Longidaza^®^ was assessed with scanning electron microscopy. The biofilms were established by seeding the bacterial suspension in BM broth in 34 mm plastic adhesive Petri dishes (TC-treated, Eppendorf, 2 mL per plate) followed by 48 h growth at 37 °C under static conditions. The mature biofilms were washed with sterile PBS, filled with fresh BM broth containing 750 IU Longidaza^®^, and incubation followed for the next 24 h. Next, the plates were washed 3 times with water and fixed with glutaraldehyde (1% water solution) for 24 h. After the subsequent washing with deionized water, the plates were dried for 12 h at 55 °C and coated in a vacuum with gold on an SCD 004 (Balzers AG, Balzers, Liechtenstein). From the each sample, 10 fields of view were analyzed. The SEM was performed on a Quanta 200 microscope (FEI Company, Hillsboro, OR, USA) at 29 kV at the Shared Research Facilities for Physical and Chemical Ultramicroanalysis, Limnological Institute of the Siberian Branch of the Russian Academy of Sciences, Irkutsk.

### 2.7. Statistical Analysis

The experiments were carried out in biological triplicates (i.e., newly prepared cultures and medium) with three independent repeats in each one. The statistical significance of the results was assessed using the Kruskal–Wallis statistical test, with a significance threshold at *p* < 0.05.

## 3. Results

### 3.1. The Effect of Longidaza^®^ on the Bacterial Biofilms In Vitro

While various strategies have been offered to date for the targeting of topical biofilms for their destruction or the prevention of their formation [31,32,33,34], very few options are available for the treatment of urinary and urogenital biofilm-associated infections. One of the promising strategies for the destruction of already-formed biofilms seems to be their enzymatic treatment.

We investigated whether Longidaza^®^—a registered drug produced by NPO Petrovax Pharma LLC, formed of bovine hyaluronidases fused with a copolymer of 1,4-ethylenepiperazine N-oxide and (N-carboxymethyl)-1,4-ethylenepiperazinium bromide (Longidaza^®^) (bovhyaluronidaze azoximer, Longidaza^®^) [56]—is capable of disrupting bacterial biofilms formed by various Gram-positive (*S. aureus* and *E. faecalis*) and Gram-negative (*E. coli*, *P. aeruginosa*, *K. pneumoniae*, *S. marcescens*) bacteria commonly causing urinary tract infections [9]. For that, bacteria were grown in BM broth for 48 h on 24-well TC-treated plates; next, the plates were washed twice by fresh BM and filled with fresh BM broth containing Longidaza^®^ at concentrations of 85, 190, 375 and 750 IU, respectively. After either 4 or 24 h incubation, the culture liquid was discarded, and the residual biofilms were quantified by crystal violet staining. The control wells were subjected to the same procedures, except for the addition of Longidaza^®^, and the absorbance in these wells was considered to be 100%.

**Figure 1:**
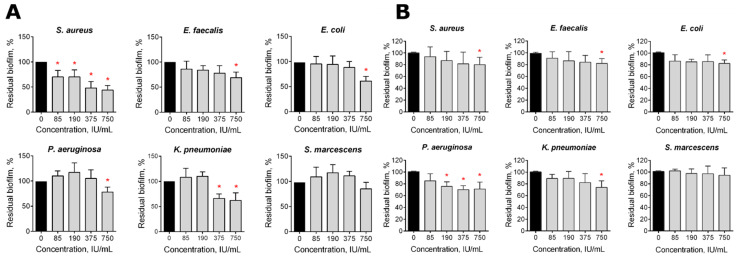
The effect of the Longidaza^®^ treatment on bacterial biofilms. The 48-h old biofilms were gently washed with BM broth, and a fresh BM broth containing Longidaza^®^ at concentrations of 85–750 IU/mL was loaded. After either 4 (**A**) or 24 (**B**) hours of treatment, the residual biofilms were quantified by crystal violet staining. The asterisks (*) denote a statistically significant difference of the biofilm in the untreated wells and wells treated with Longidaza^®^ (*p* < 0.05).

The data shown in Figure 1 indicate that Longidaza^®^ is capable of destroying established 2-day-old biofilms formed by almost all of the studied bacteria after four hours of treatment. The maximum effect could be observed for *S. aureus* biofilms, with a biofilm biomass reduction of 20% at 85 IU, and of 50% at 750 IU/mL of the enzyme (Figure 1A). The significant destruction of biofilms of *E. faecalis*, *E. coli, P. aeruginosa* and *K. pneumoniae* was observed after 4 h exposure to 750 IU/mL Longidaza^®^, with the residual biofilms containing 60–70% of their initial biomass, while no significant effect on the biofilm of *S. marcescens* could be observed. The effect after 24 h of treatment was less pronounced, apparently because of the enzyme inactivation and/or damage (Figure 1B). Nevertheless, 15–20% biofilm biomass reduction could be observed for all of the bacteria (excluding *S. marcescens*) at the drug concentration of 750 IU/mL.

**Figure 2:**
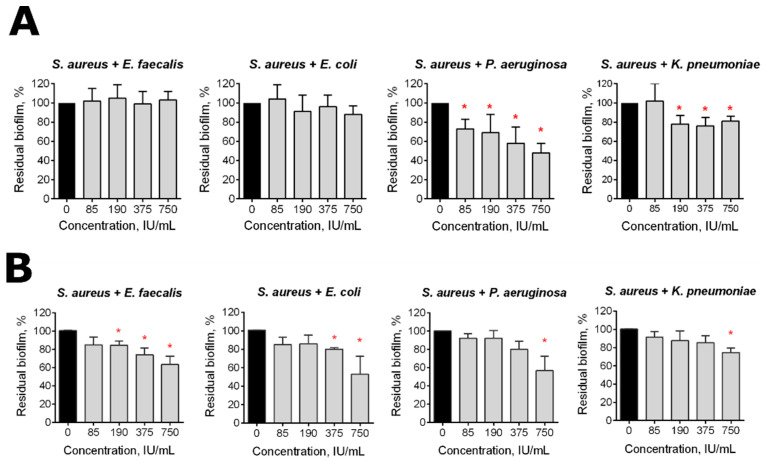
The effect of Longidaza^®^ on mixed bacterial biofilms. The 48-h old biofilms were gently washed with BM broth, and a fresh BM broth containing Longidaza^®^ at concentrations of 85–750 IU/mL was loaded. After either 4 (**A**) or 24 (**B**) hours of treatment, the residual biofilms were quantified by crystal violet staining. The asterisks (*) denote statistically a significant difference of the biofilm in the untreated wells and wells treated with Longidaza^®^ (*p* < 0.05).

In many cases, several species composing a microbial consortium while containing only one pathogenic bacterium, altogether contribute to the development of long-term infectious diseases [6]. *S. aureus* has been reported to form mixed biofilms with various Gram-negative bacteria [16,20,23]. In order to test the effect of Longidaza^®^ on mixed bacterial biofilms, *S. aureus* was inoculated with either *E. faecalis*, *E. coli, P. aeruginosa* or *K. pneumoniae* and grown for 48 h to obtain dual-species biofilms, which were subsequently treated with Longidaza^®^ for either 4 or 24 h. As can be seen from Figure 2, a significant reduction of the residual biofilm was observed in all of the bacterial combinations after 24 h treatment with the enzyme. Moreover, a 4-h treatment of mixed communities with Longidaza^®^ also lead to a significant reduction of biofilms formed by *S. aureus–P. aeruginosa* and *S. aureus–K. pneumoniae*, suggesting that Longidaza^®^ could be a promising tool for bacterial biofilm destruction.

### 3.2. Scanning Electron Microscopy

**Figure 3:**
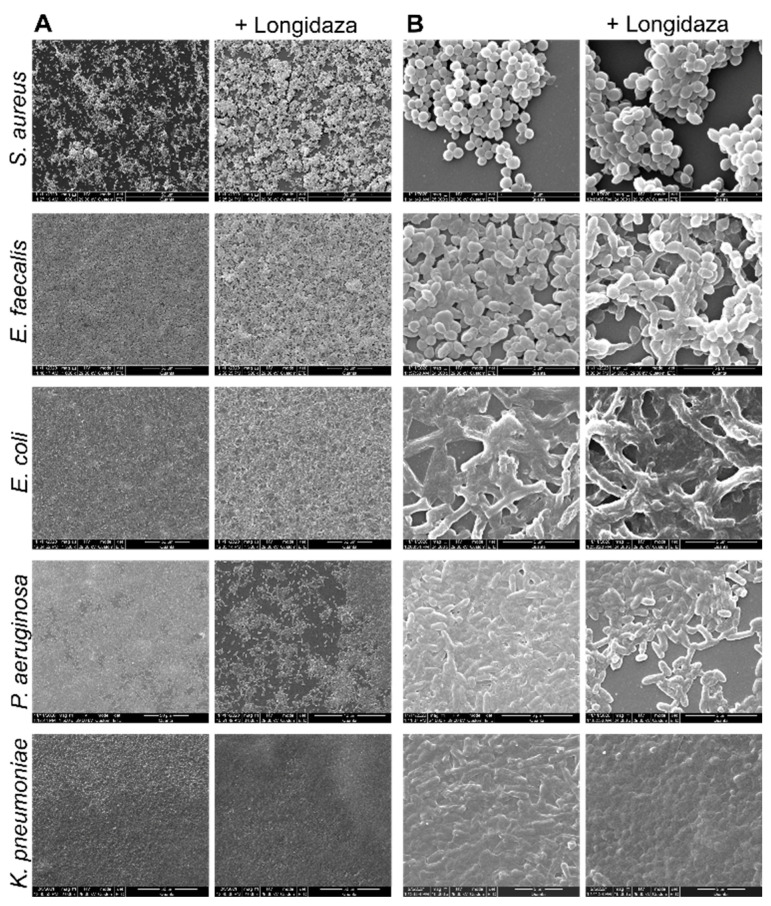
Scanning electron microscopy of 48-h old biofilms of *S. aureus*, *E. faecalis, E. coli, P. aeruginosa* and *K. pneumoniae,* treated for 24 h with Longidaza^®^ at concentration of 750 IU/mL. Magnification 1500× (**A**) and 24,000× (**B**).

**Figure 4:**
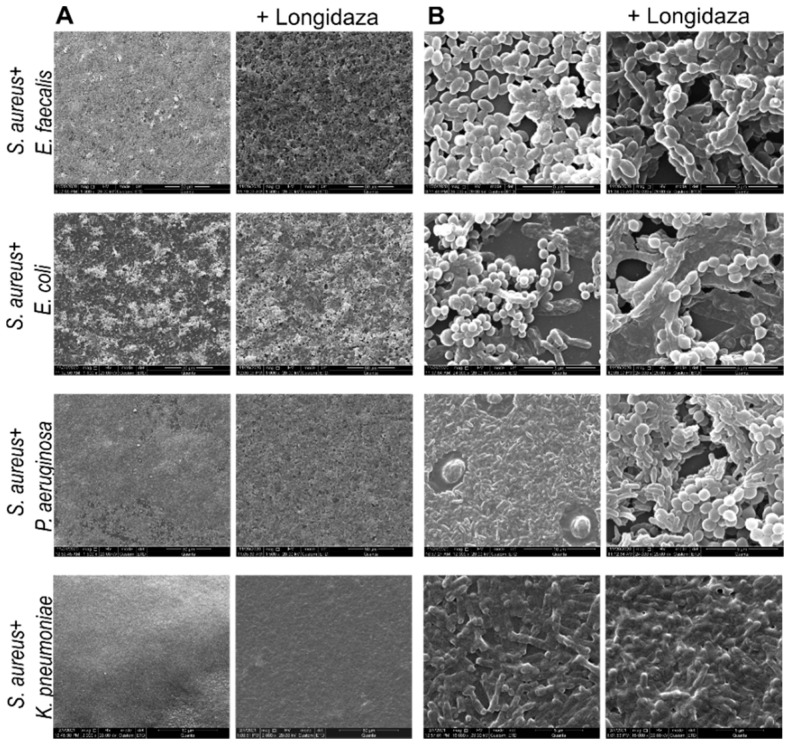
Scanning electron microscopy of 48-h old mixed biofilms of *S. aureus* with either *E. faecalis, E. coli, P. aeruginosa* or *K. pneumoniae,* treated for 24 h with Longidaza^®^ at concentration of 750 IU/mL. Magnification 1500× (**A**) and 24,000× (**B**).

In order to visualize the effect of Longidaza^®^ on the biofilm’s structure, 48-h old biofilms of *S. aureus, E. faecalis, E. coli, P. aeruginosa* and *K. pneumoniae* were treated for 24 h as previously with the enzyme, fixed with glutaraldehyde and analyzed using scanning electron microscopy. As can be seen from Figure 3, treatment with Longidaza^®^ had hardly any effect on the visual structure of *S. aureus* and *K. pneumoniae* biofilms. By contrast, the formation of pores and cavities could be clearly observed in the treated biofilms of *E. faecalis, E. coli,* and *P. aeruginosa*, in comparison with the dense, multilayer structures visualized for the control wells. The most pronounced effect could be observed for *P. aeruginosa*, where the biofilm was reduced down to a monolayer of adherent cells and even single cells. Similar effects were detected for mixed biofilms of *S. aureus* with either *E. faecalis, E. coli* or *P. aeruginosa* (Figure 4). Thus, the treated biofilms of these consortia exhibited a pronounced porous structure in comparison with the untreated wells. Again, no significant effect could be observed for the *S. aureus–K. pneumoniae* dual-species biofilm, which is in agreement with the less pronounced effect observed in the crystal-violet stain experiments.

### 3.3. The Effect of Longidaza^®^ on the Structural Components of Bacterial Biofilms

**Figure 5:**
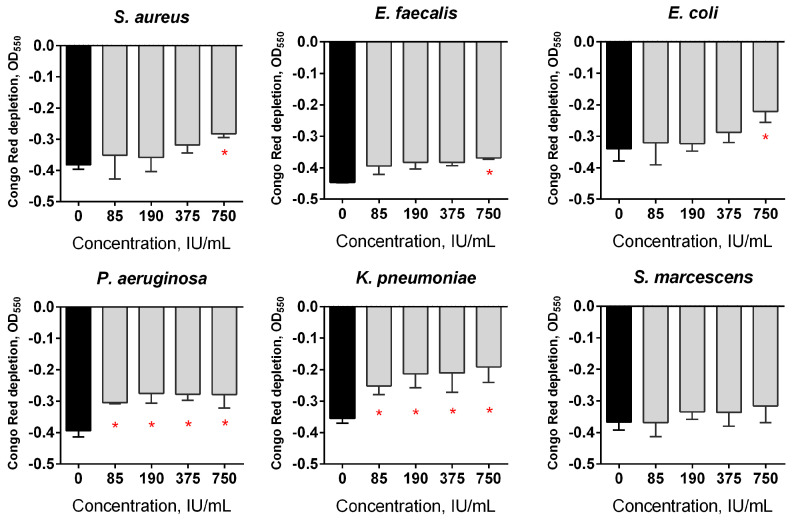
The effect of Longidaza^®^ treatment on bacterial biofilms. The 48-h old biofilms were gently washed with BM broth, and a fresh BM broth containing Longidaza^®^ at concentrations of 85–750 IU/mL was loaded. After 24 h of treatment, the residual biofilms were quantified using a Congo Red depletion assay. The asterisks (*) denote a statistically significant difference of the biofilm in the untreated wells and the wells treated with Longidaza^®^ (*p* < 0.05).

**Figure 6:**
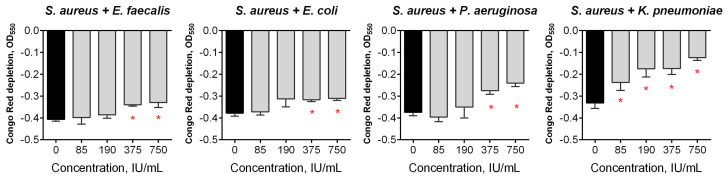
The effect of Longidaza^®^ treatment on mixed bacterial biofilms. The 48-h old biofilms were gently washed with BM, and a fresh BM broth containing Longidaza^®^ at concentrations of 85–750 IU/mL was loaded. After 24 h of treatment, the residual biofilms were quantified using a Congo Red depletion assay. The asterisks (*) denote a statistically significant difference of the biofilm in the untreated wells and the wells treated with Longidaza^®^ (*p* < 0.05).

In order to assess the effect of Longidaza^®^ on the components of the biofilm matrix, a Congo red depletion assay was performed. Treatment with Longidaza^®^ for 24 h led to the reduction of the Congo red uptake in the biofilms of all of the studied bacteria, with the exception of *S. marcescens*. Of note, a more pronounced effect of Longidaza^®^ assessed by Congo red staining was observed for Gram-negative bacteria (*E. coli, P. aeruginosa, K. pneumoniae*) (Figure 5). In mixed biofilms, a more pronounced effect was observed for consortia of *S. aureus* with either *P. aeruginosa* or *K. pneumoniae* (Figure 6).

In order to gain further insight into the components of the biofilm that are destroyed by Longidaza^®^, the biochemical composition of the extracellular matrix was analyzed with the differential fluorescent staining of the biomolecules. For this purpose, mature 48-h old biofilms were treated with Longidaza^®^ (750 IU/mL) and stained with Concanavalin A, Calcofluor white, and Sypro Orange to evaluate the changes of the α-polysaccharides, β-polysaccharides and proteins in the biofilm matrix, respectively. As a control, intact biofilms of the same bacteria were stained. In agreement with the crystal violet and Congo Red assays, a significant reduction of β-polysaccharides in the biofilms of *S. aureus, E. faecalis, E. coli* and *P. aeruginosa* was observed after the enzymatic treatment (Figure S1), suggesting the preferred hydrolysis of β-glycosyl bonds by Longidaza^®^. Nevertheless, in the *E. faecalis* biofilm, a significant decrease of α-polysaccharides could also be observed (Figure S1), with the latter effect possibly being attributable to the general biofilm destruction.

Thus, Longidaza^®^ promotes the hydrolysis of β-polysaccharides in the matrix of biofilms of the studied bacteria, leading to their subsequent destruction. Of note, the observed effect varies between species, which is apparently governed by the features of each bacterium or their consortia.

### 3.4. The Effect of Longidaza^®^ on the Efficacy of Antimicrobials against Biofilm-Embedded Bacteria

**Figure 7:**
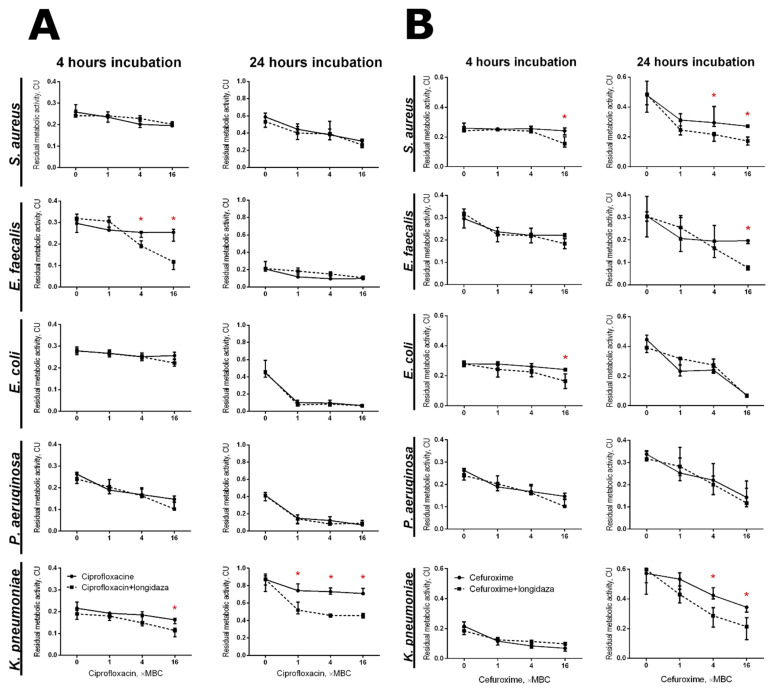
The effect of Longidaza^®^ on the susceptibility of biofilm-embedded bacteria to antimicrobials. Longidaza^®^ was added to 48-h-old biofilms to a final concentration of 750 IU/mL. Ciprofloxacin (**A**) and cefuroxime (**B**) were added up to final concentrations of 1–16× MBC (see Table S1 for the values). After 24 h incubation, the biofilms were washed twice with sterile 0.9% NaCl. The viability of the adherent cells was analyzed with an MTT assay. The asterisks (*) denote a statistically significant difference of the residual respiratory activity in the untreated wells (solely antimicrobials) and wells with the combined treatment (*p* < 0.05).

While embedded into the biofilm matrix, bacterial cells become largely inaccessible to both antibiotics and biocides. The above data suggest that biofilm matrix destruction with Longidaza^®^ could facilitate the penetration of antimicrobials into biofilm-embedded bacteria, as has been shown previously in other model investigations [33,34,35,66]. For this, 48-h old biofilms were incubated for either 4 or 24 h in the presence of Longidaza^®^ (750 IU) with antimicrobials (Ciprofloxacine and Cefuroxime) at their respective 1×, 4× and 16× MBCs (minimal bactericidal concentrations, see Table S1 for values), followed by the evaluation of the biofilm-embedded cells’ viability with an MTT-assay (Figure 7). After 4 h of treatment of *E. faecalis* biofilms with Ciprofloxacine in combination with Longidaza^®^, a 3.5-fold drop of cell viability was observed, while solely the antimicrobial did not have any significant effect (Figure 7A). For *E. coli*, *P. aeruginosa* and *K. pneumoniae,* the effect was less pronounced. After 24 h of treatment, the viability of *E. faecalis, E. coli* and *P. aeruginosa* decreased significantly, irrespective of the treatment with Longidaza^®^, while Ciprofloxacine could affect the *K. pneumoniae* viability only in the presence of the enzyme. The efficacy of Ciprofloxacine against *S. aureus* could not be improved by combination with Longidaza^®^. By contrast, the combination of Longidaza^®^ with Cefuroxime demonstrated synergy on *S. aureus* (Figure 7B) after both 4 and 24 h treatment, as well as with *E. faecalis* and *K. pneumoniae* after 24 h treatment. At the same time, no significant increase of the antibiotic efficacy on *P. aeruginosa* biofilm-embedded cells was detected. In a qualitative metabolic test with rezazurine, a significant increase of Cefuroxime efficacy was detected only on *E. faecalis* biofilms (Figure S2). On the other hand, the nearly complete eradication of *S. aureus, E. faecalis* and *P. aeruginosa* detached cell clumps could be observed after treatment with a combination of Longidaza^®^ with 1× MBC of Cefuroxime, while solely the antibiotic remained inefficient even at 16× MBC (Figure S2).

Quite surprisingly, no synergistic effect of Longidaza^®^ with antimicrobials could be observed for dual-species biofilms in the MTT assay, where solely antibiotic treatment already led to a significant reduction of the cells’ viability (Figure S3). Again, the resazurine test revealed an increased efficacy of Cefuroxime against cell clumps detached from dual-species biofilms formed by *S. aureus* in combination with *E. faecalis, E. coli* or *P. aeruginosa* (Figure S4). Additionally, the moderate enhancement of the treatment efficacy against *S. aureus*–*E. faecalis* mixed biofilms could also be observed. No significant effect was detected for Ciprofloxacine. The latter fact could be plausibly attributed to the complex interactions of *S. aureus* with other bacteria in co-cultures, resulting in their drastically altered susceptibility to antimicrobials, as has been shown for aminoglycosides and Ciprofloxacine [67,68], although unraveling the particular mechanisms governing these interactions requires further investigation.

## 4. Discussion

Since ancient times, improving the effectiveness of the treatment of infections has remained an important challenge in clinical medicine. Biofilm formation on tissues and catheters is an important factor of bacterial virulence. An extracellular biofilm matrix effectively shields bacteria from the immune system of the host as well, as from antimicrobials, providing up to 1000-fold higher tolerance to antibiotics compared to their planktonic forms [69,70,71]. In urinary patients, both implanted catheters and the epithelium of the urinary tract are subjected to bacterial biofilm fouling, in turn leading to the development of long-term diseases [6,17,18] such as chronic bacterial prostatitis and pyelonephritis [20,21]. While the enzymatic destruction of biofilms in general seems to be an attractive approach [31,32,33,34,35,43,47,50,51], relatively few enzymes have been proposed for the treatment of urinary biofilms. In clinical practice, most enzymes are used for wound healing, and are not suitable for internal use due to the low stability of the preparations, as well as the high allergic potential [72,73,74]. Longidaza^®^ is already used in clinical practice, and in some reports the application of the enzyme led to the increased detection of microbial contamination in the semen and urea of patients with various diseases of the urogenital tract [57,58,59,60].

Here, we show in vitro the antibiofilm activity of Longidaza^®^, a bovine hyaluronidase fused with a copolymer of 1,4-ethylenepiperazine N-oxide and (N-carboxymethyl) -1,4-ethylenepiperazinium bromide, which stabilizes the enzyme and increases its activity [56]. Hyaluronic acid, a large glycosaminoglycan, has been reported to make an essential contribution to the formation of staphylococcal biofilms. In turn, the destruction of hyaluronic acid with hyaluronidase led to the effective destruction of staphylococcal biofilms [54,55]. A similar effect has been shown for *Streptococcus intermedius*, which splits hyaluronic acid for the initial adhesion while forming a consortium on the surface of tissues [55]. In accordance with these data, the significant destruction of staphylococcal biofilm with Longidaza^®^ has been observed after 4 h of treatment (Figure 1A). The biofilm biomasses of other bacteria were also diminished, although with lower efficiency. Of note, after 24 h of treatment, the biofilm destruction was less pronounced (Figure 1B), apparently due to the inactivation of the enzyme [75,76,77]. By contrast, in mixed cultures, 24 h treatment led to biofilm biomass reduction by 30–50%, while 4 h treatment did not affect *S. aureus*-*E. faecalis* and *S. aureus*-*E. coli* biofilms (Figure 2). In previous studies, the most fascinating results were observed for proteases for the targeting of staphylococcal biofilms [35,44,45,46], while glycoside hydrolases were effective against gram negative bacteria [47,48,49,50,51]. According to our results, Longidaza^®^ promotes the destruction of monospecies biofilms of both Gram-positive and Gram-negative bacteria, as well as their mixed communities, suggesting Longidaza^®^ as a promising enzyme for the combating of biofilm-associated polymicrobial infections. Of note, the biomass of a *S. aureus–P. aeruginosa* mixed biofilm was reduced twofold after 4 h treatment, which makes Longidaza^®^ a promising tool to improve the treatment of acute and chronic wounds, as well as in cystic fibrosis complicated by *S. aureus–P. aeruginosa* mixed biofilm formation [78,79,80,81,82,83,84,85].

The in vitro ability of Longidaza^®^ to destroy the biofilms formed by various bacteria fits with recent clinical data reporting Longidaza^®^ to increase the frequency of pathogens’ detection in the urogenital tract of patients [57,58,59,60,86], apparently via the destruction and dispersion of biofilms. Indeed, the differential staining of the components of intact and treated biofilms revealed a significant reduction of β-polysaccharides, one of the main components of the biofilm matrix [53]. On the other hand, the appearance of bacteria in urea and semen after Longidaza^®^ treatment [86] suggests the necessity of the combination of Longidaza^®^ with antimicrobials to prevent bacteremia.

Accordingly, the combination of Longidaza^®^ with antimicrobials, due to its capability of destroying the biofilm matrix, appears to be a promising direction for the further improvement of antimicrobial treatment, as supported by both recent clinical data [57,58,59,60] and the in vitro observations of this study (Figure 7). The efficacy of ciprofloxacine and cefuroxime was significantly enhanced, particularly against *S. aureus, E. faecalis, E. coli, P. aeruginosa* and *K. pneumoniae* monomicrobial biofilms, although the synergy of the enzymatic and antimicrobial treatment varied for different bacteria and treatment times. Apparently, this could be attributed to the variations in the biofilm matrix composition formed by various bacteria, which in turn affects their susceptibility to different antimicrobials [53]. In particular, it has been shown that cellulose and the amyloid protein ‘curly’ are the main components of the biofilm matrix of enterobacteria, while the main components of the biofilm matrix formed by *E. faecalis*, *P. aeruginosa*, and *K. pneumoniae* biofilms are polysaccharides and eDNA [87,88,89]. Additionally, Ciprofloxacin has been reported to penetrate the biofilms of the majority of bacteria [90,91], which apparently decreases the further effect of the matrix destruction by Longidaza^®^.

Surprisingly, no significant increase of antimicrobial efficacy in the presence of Longidaza^®^ has been observed for dual-species biofilms formed with *S. aureus* and other tested bacteria (Figure S3). Because solely antibiotic treatment led to a significant decrease of the cells’ viability, such an effect could be a consequence of complicated interactions of *S. aureus* with other bacteria in mixed biofilms that result in their altered susceptibility to antimicrobials [67,68]. Notably, several recent investigations have reported that various metabolites produced by *P. aeruginosa* increase the sensitivity of *S. aureus* biofilms to fluoroquinolones, membrane-targeting antibacterial agents and antiseptic chloroxylenol, while simultaneously promoting their tolerance to beta-lactams, glycopeptides, aminoglycosides and macrolides [92,93,94]. In turn, *S. aureus* also affects the susceptibility of *P. aeruginosa* to antibiotics in biofilms [67]. Nevertheless, the synergetic effect was visible in the rezazurine test on cell clumps detached from the biofilm (Figure S4), an intermediate state between biofilm and planktonic cells [95], confirming that, despite of the above limitations, Longidaza^®^ could be an efficient tool for the treatment of biofilm-associated infection.

Taken together, our data indicate that the combination of antimicrobial treatment with Longidaza^®^ could significantly increase the effectiveness of biofilm-associated infections treatment, although an additional analysis to reveal the antimicrobials exhibiting pronounced synergy with the enzyme is required to achieve the best treatment outcomes.

## 5. Conclusions

Taken together, our results indicate that Longidaza^®^ destroys the monomicrobial and mixed biofilms formed by Gram-positive and Gram-negative bacteria, which represent the major causes of catheter-associated and urogenital infections. Scanning electron microscopy confirmed the reduction of the biofilm biomass and the formation of porous structures in both monomicrobial and mixed communities after treatment with Longidaza^®^. Consequently, the combined use of the enzyme with antimicrobials promotes the effect of the latter against bacteria within biofilms. Thus, we believe that Longidaza^®^ could serve as an effective tool to target both external and internal infections associated with monomicrobial and mixed biofilms.

## Figures and Tables

**Table 1:** The wavelengths of the emission and excitation of the fluorescent dye compared to the components of the biofilm staining.

Dye	ConA-TMR	CFW	Sypro Orange
Excitation Wavelength	552 nm	254 nm	470 nm
Emission Wavelength	578 nm	432 nm	570 nm
Target	α-polysaccharides	β-polysaccharides	Proteins

## Data Availability

All data are present in the manuscript.

## Supplementary Materials

The following are available online at https://www.mdpi.com/article/10.3390/pharmaceutics13111740/s1, Table S1: Minimum Inhibitory Concentration (MIC) and Minimal Bactericidal Concentration (MBC) values of the antimicrobials. Figure S1: Differential evaluation of the fractions of the biofilm matrix components of various bacteria before and after 24 h treatment with Longidaza^®^. Mature 48 h old biofilms were treated with Longidaza^®^ (750 IU/mL) and stained with Concanavalin A, Calcofluor, and SyproOrange to evaluate the changes of the α-polysaccharides, β-polysaccharides and protein composition, respectively. The asterisks (*) denote a statistically significant difference of the fluorescence in the untreated wells and wells treated with Longidaza^®^ (*p* < 0.05). Figure S2: The effect of Longidaza^®^ on the susceptibility of biofilm-embedded bacteria to antimicrobials. Longidaza^®^ was added to 48-h-old biofilms until a final concentration of 750 IU/mL. Ciprofloxacin and cefuroxime were added up to final concentrations of 1–16× MBC (see Table S1 for values). After 24 h incubation, the biofilms were washed twice with sterile 0.9% NaCl. The viability of the adherent cells was analyzed using a resazurine test. The blue boxes denote wells with non-viable cells. Figure S3: The effect of Longidaza^®^ on the susceptibility of biofilm-embedded dual-species bacterial consortia to antimicrobials. Longidaza^®^ was added to 48-h-old biofilms until a final concentration of 750 IU/mL. Ciprofloxacin and cefuroxime were added up to final concentrations of 1–16× MBC (see Table S1 for values). After 24 h incubation, the biofilms were washed twice with sterile 0.9% NaCl and the viability of adherent cells was analyzed using an MTT assay. The asterisks (*) denote a statistically significant difference of the residual respiratory activity in untreated wells (solely antimicrobials) and wells with a combined treatment (*p* < 0.05). Figure S4: The effect of Longidaza^®^ on the susceptibility of biofilm-embedded dual-species bacterial consortia to antimicrobials. Longidaza^®^ was added to 48-h-old biofilms to a final concentration of 750 IU/mL. Ciprofloxacin and cefuroxime were added up to final concentrations of 1–16× MBC (see Table S1 for values). After 24 h incubation, the biofilms were washed twice with sterile 0.9% NaCl, and the viability of the adherent cells was analyzed using a resazurine test. The blue boxes denote wells with non-viable cells.
