# Supplementary material for: Improving the Efficacy of Antimicrobials against Biofilm-Embedded Bacteria Using Bovine Hyaluronidase Azoximer (Longidaza®)
Source: Pharmaceutics. 2021 Oct 20;13(11):1740. doi: 10.3390/pharmaceutics13111740 (PMC8622991; doi:10.3390/pharmaceutics13111740)
Supplement: Supplementary file 1 [file pharmaceutics-13-01740-s001.zip › pharmaceutics-1414396-supplementary (1).pdf]

pharmaceutics

MDPI

# Supplementary Materials:Improving the Efficacy of Antimicrobials against Biofilm-Embedded Bacteria using Bovine Hyaluronidase Azoximer (Longidaza®)

**Elena Trizna, Diana Baydamshina, Anna Gorshkova, Valentin Drucker, Mikhail Bogachev, Anton Tikhonov and Airat Kayumov**

**Table S1.** Minimum Inhibitory Concentration (MIC) and Minimal Bactericidal Concentration (MBC) values of antimicrobials.

| | Ciprofloxacin | | Cefuroxime | |
|---|---|---|---|---|
| | MIC, μg/mL | MBC, μg/mL | MIC, μg/mL | MBC, μg/mL |
| *S. aureus* | 0.5 | 32 | 8 | 64 |
| *E. faecalis* | 8 | 32 | 8 | 32 |
| *E. coli* | 0.5 | 1 | 4 | 8 |
| *K. pneumonia* | 0.25 | 16 | 8 | 64 |
| *P. aeruginosa* | 0.5 | 1 | 8 | 64 |

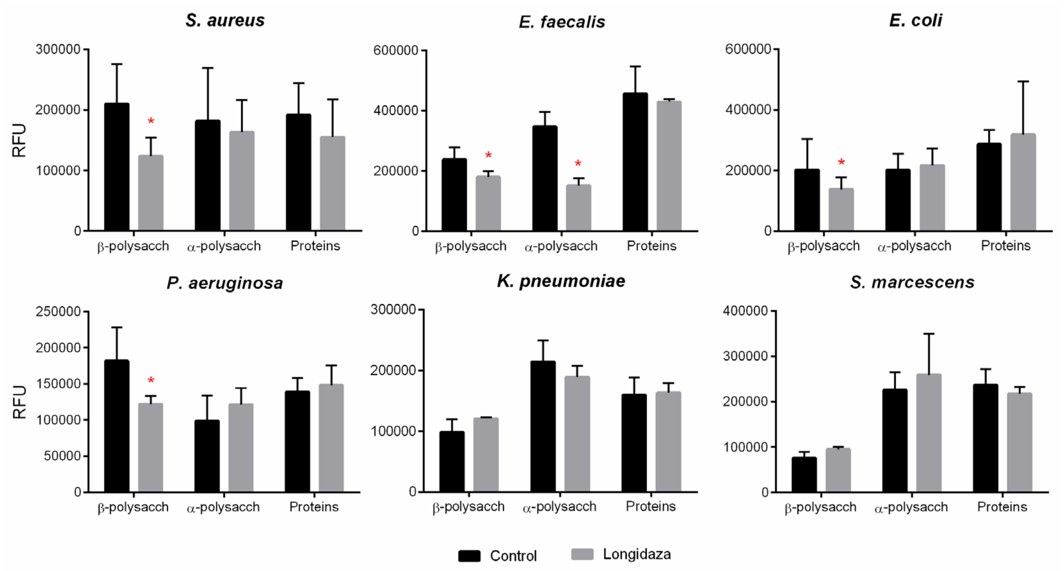


**Figure S1.** Differential evaluation of the fractions of biofilm matrix components of various bacteria before and after 24 h treatment with Longidaza®. Mature 48 h old biofilms were treated with Longidaza® (750 IU/mL) and stained with Concanavalin A, Calcofluor, and SyproOrange to evaluate the changes of α-polysaccharides, β - polysaccharides and proteins composition, respectively. Asterisks (*) denote statistically significant difference of fluorescence in untreated wells and wells treated with Longidaza® ($p < 0.05$).The text continues here (Figure 2 and Table 2).

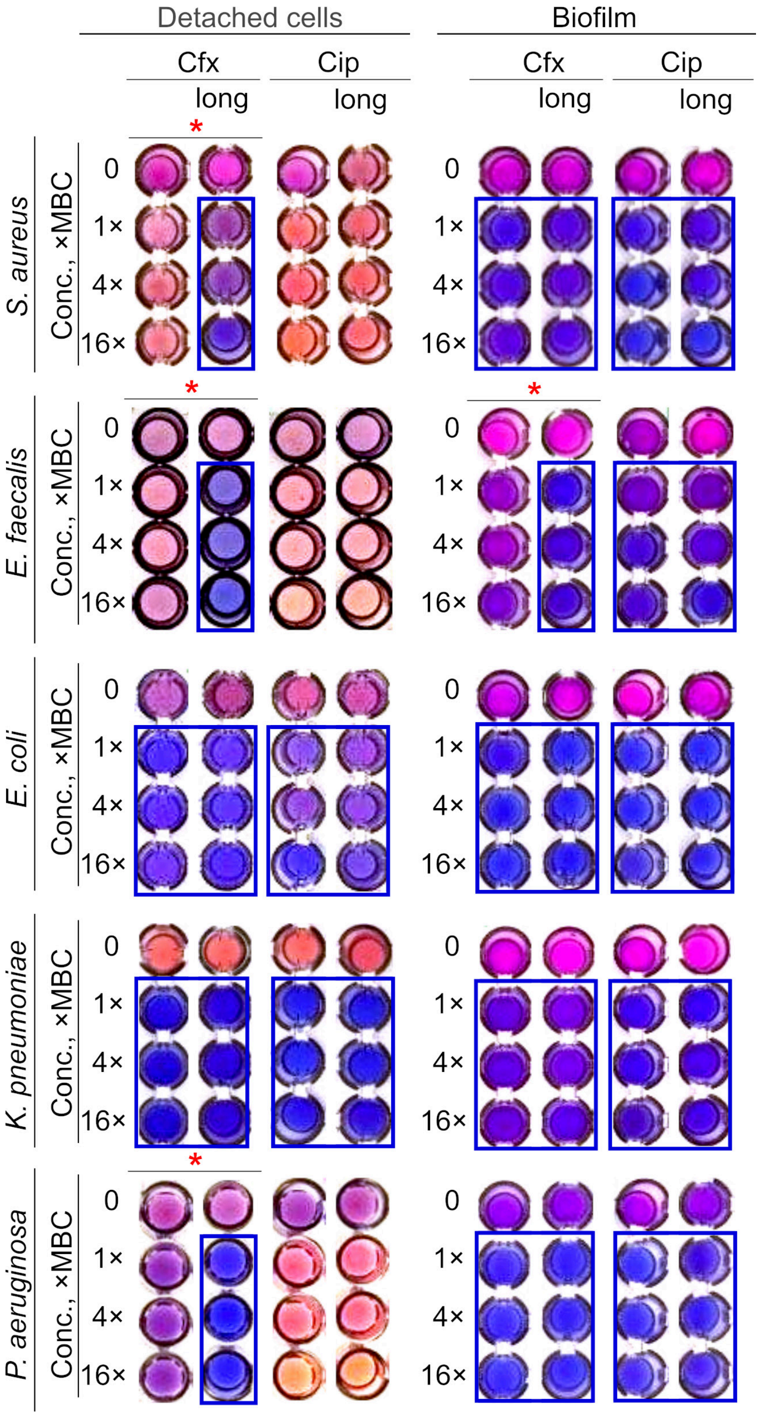


**Figure S2.** The effect of Longidaza® on the susceptibility of biofilms-embedded bacteria to antimicrobials. Longidaza® was added to 48 h old biofilms until final concentration of 750 IU/mL. Ciprofloxacin and cefuroxime were added up to final concentrations of 1-16× MBC (see Table S1 for values). After 24 h incubation, the biofilms were washed twice with sterile 0.9% NaCl. The viability of adherent cells was analyzed with resazurine test. Blue boxes denote wells with non-viable cells. * shows where there was a difference between treated and untreated wells.

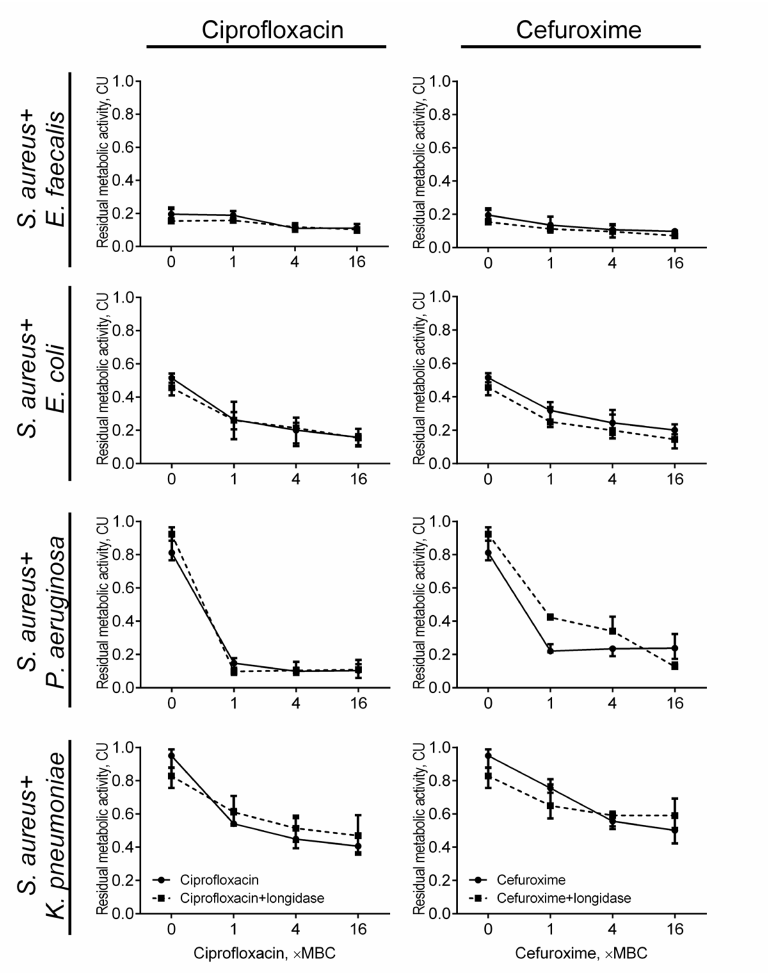


**Figure S3.** The effect of Longidaza® on the susceptibility of biofilms-embedded dual species bacterial consortia to antimicrobials. Longidaza® was added to 48 h old biofilms until final concentration of 750 IU/mL. Ciprofloxacin and cefuroxime were added up to final concentrations of 1-16×MBC (see Table S1 for values). After 24 h incubation, biofilms were washed twice with sterile 0.9% NaCl and the viability of adherent cells was analyzed with MTT assay.

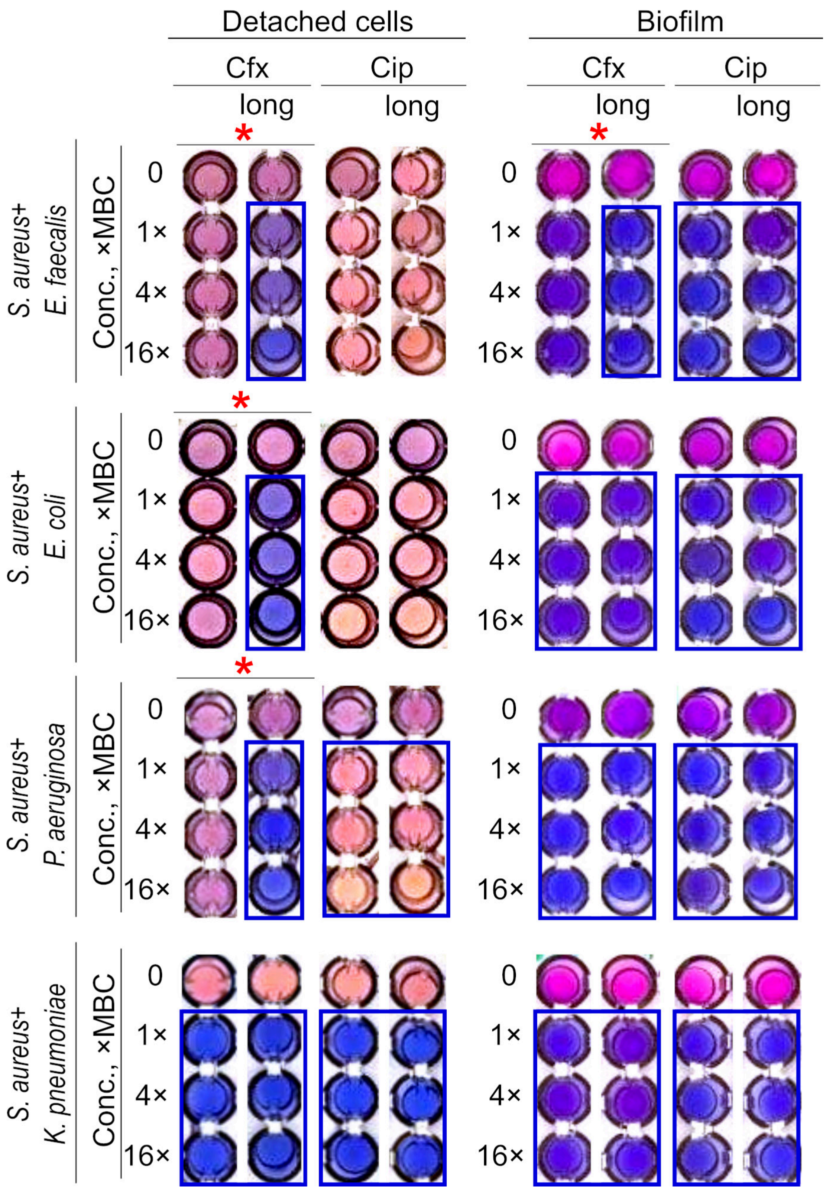


**Figure S4.** The effect of Longidaza® on the susceptibility of biofilms-embedded dual species bacterial consortia to antimicrobials. Longidaza® was added to 48 h old biofilms until final concentration of 750 IU/mL. Ciprofloxacin and cefuroxime were added up to final concentrations of 1-16×MBC (see Table S1 for values). After 24 h incubation, biofilms were washed twice with sterile 0.9% NaCl and the viability of adherent cells was analyzed with resazurine test. Blue boxes denote wells with non-viable cells. * shows where there was a difference between treated and untreated wells.
